# Evidence for the benefits of lifestyle medicine interventions in breast cancer survivorship

**DOI:** 10.1093/oncolo/oyae303

**Published:** 2024-12-03

**Authors:** Laura E Wright, Preeti K Sudheendra

**Affiliations:** Department of Internal Medicine, The Ohio State University Medical Center, Columbus, OH, United States; The James Cancer Hospital and Solove Research Institute, The Ohio State University Medical Center, Columbus, OH, United States

**Keywords:** breast cancer, survivorship, lifestyle medicine, psychosocial health, cancer recurrence

## Abstract

There are currently 4 million breast cancer survivors in the United States, and this number is expected to substantially increase in the decades to come. Breast cancer survivors experience treatment- and cancer-related debility, stress, and isolation that exceed rates in the general population. This review provides evidence for survival and quality of life benefits in patients living with breast cancer with the implementation of the 6 pillars of lifestyle medicine, which include physical activity, nutrition, social connection, adequate sleep, stress management, and avoidance of toxic substances. Overwhelmingly, lifestyle modifications and support of psychosocial health improve survival and quality of life in patients living with breast cancer. Data presented here suggest that patients living with breast cancer would benefit from a comprehensive lifestyle medicine approach to survivorship and formal implementation of such programs could significantly impact cancer mortality and morbidity.

**Implications for Practice**

The number of breast cancer survivors is increasing year-over-year, and these patients often experience recurrence and treatment-related debility, stress, and isolation. There is substantial evidence that lifestyle medicine interventions improve disease-free survival and reduce all-cause mortality. The implementation of Cancer Center-sponsored lifestyle medicine programs aimed at improving social connectivity, sleep, nutrition, and exercise, and reducing the harms of stress and toxins in patients living with breast cancer has great potential to improve morbidity and mortality. Access to formal lifestyle medicine programs should be available for all patients with a breast cancer history.

## Introduction

Breast cancer is the most common cancer affecting women with an estimated 4 million survivors currently residing in the United States.^1^ The number of breast cancer survivors is increasing year-over-year due to a decline in breast cancer mortality in the setting of early detection and improved treatments. Despite living longer, breast cancer survivors experience treatment-induced and cancer-related debility, stress, isolation, and sleep disturbances at rates that tend to exceed that of the general population.^2,3^ The field of lifestyle medicine has emerged over the past 20 years with the purpose of enabling patients to adopt and sustain behaviors that improve health and quality of life.^4^ The 6 pillars of lifestyle medicine are (1) social connection, (2) physical activity, (3) whole food and plant-based nutrition, (4) stress management, (5) restorative sleep, and (6) avoidance of risky behaviors or toxins (Table 1). In line with the goals of implementing these 6 pillars, the American Society of Clinical Oncology (ASCO) has put forth guidelines for cancer survivorship that include proactive management of physical and psychosocial effects of breast cancer and breast cancer treatment with the adoption of healthy lifestyle habits.^5^ Integrative medicine strategies that enhance social connections, physical activity, nutrition, sleep, stress management, and avoidance of unhealthy habits can play an integral role in optimizing breast cancer survivorship. To date, there is no comprehensive review of each of the lifestyle medicine pillars and their relationship to issues of breast cancer survivorship. Presented here is a summary of the evidence for the benefit of incorporating each of the lifestyle medicine pillars into cancer survivorship to improve quality of life and decrease the risk of cancer recurrence and mortality.

**Table 1.** Six pillars of lifestyle medicine.

1. Physical activity
2. Whole food and plant-based nutrition
3. Social connections
4. Restorative sleep
5. Stress management
6. Avoidance of risky behaviors and substances

## Physical activity

Surgery, radiation, chemotherapy, and endocrine therapy have side effects including pain, debility, cardiotoxicity, arthralgias, and weight gain. These adverse effects are largely attributable to estrogen deprivation, which negatively impact exercise capacity, mobility, cardiovascular function, and quality of life. Observational studies consistently point toward the mortality benefit of exercise following a cancer diagnosis. A systematic review of the literature through 2014 performed by Lahart et al looked at 22 prospective cohort studies with average follow-up periods of 4-13 years and found that both pre- and postdiagnosis physical activity were associated with reduced risk of breast cancer events, including progression, new primaries, or recurrence (hazard ratio (HR) = 0.72, 95% CI, 0.56-0.91, and HR = 0.79, 95% CI, 0.63-0.98, respectively).^6^ Spei et al confirmed these observational findings in a more recent systematic review of the literature, including 10 studies with an average follow-up ranging from 3.5 to 12.7 years.^7^ Physical activity in individuals diagnosed with early-stage breast cancer was associated with reduced risk of breast cancer-specific and all-cause mortality (HR = 0.6, 95% CI, 0.36-0.99, and HR = 0.58, 95% CI, 0.45-0.75, respectively).

In subgroup analyses, associations between recent pre- and postdiagnosis physical activity and risk of all-cause mortality did not differ by body mass index (BMI) category and longitudinal benefits were observed regardless of BMI category; however, patients with BMI ≥25 kg/m^2^ had a greater reduction in breast cancer-related death,^6^ suggesting that overweight patients may benefit most from implementation of an exercise prescription. This finding is concordant with a substantial body of literature, demonstrating that obesity is associated with an increased risk of breast cancer and disease recurrence.^8^ Additionally, the associations between physical activity and risk of all-cause and breast cancer-related death were stronger for postmenopausal breast cancer survivors relative to premenopausal survivors.^6^

A systematic review of moderate-intensity exercise interventions with a mean duration of 19 weeks with 3 sessions per week in breast cancer patients and survivors showed a clear benefit in cardiorespiratory fitness, strength, fatigue, and health-related quality of life.^9^ The evidence for the benefit of exercise in cancer populations is robust enough that the American College of Sports Medicine convened a multidisciplinary roundtable in 2018 to set recommendations for the prescription and delivery of exercise in patients living with cancer.^10^ The consortium statement concluded that strong evidence exists for aerobic and resistance exercise in the treatment of anxiety, depression, fatigue, and lymphedema, and improvement in physical function and health-related quality of life in patients living with cancer. On the basis of current literature, the exercise regimen found to provide the most benefit for patients living with cancer was moderate-intensity aerobic training 3 times per week for a minimum of 8-12 weeks.^10^ The addition of resistance training to aerobic exercise at least 2 times per week results in similar benefits, and resistance training alone is efficacious at improving most health-related outcomes except for symptoms of depression where the data are less clear.^9,10^ Importantly, these studies found that supervised exercise programs were more effective than unsupervised or home-based programs, likely because a higher dose of exercise (ie, intensity and frequency) tends to be achieved in a supervised format, although other attributes of a supervised setting including motivation and reinforcement may also contribute to higher efficacy.

These results point toward a clear and significant benefit of aerobic and resistance exercise training in patients living with breast cancer and survivors and particularly for postmenopausal women with BMI ≥25 kg/m^2^. In accordance with striking evidence favoring physical activity, the ASCO formally recommends that patients living with cancer are advised to exercise during treatment to improve quality of life, reduce treatment toxicity, and control cancer progression.^11^

## Nutrition

In line with findings related to physical exercise, nutrition has been a focus of study in patients living with breast cancer because of the known link between obesity and breast cancer risk and recurrence.^8^ The American College of Lifestyle Medicine recommends a predominantly plant-based diet with a variety of minimally processed vegetables, fruits, whole grains, legumes, nuts, and seeds. A handful of prospective trials and meta-analyses have examined dietary interventions or diet quality on breast cancer outcomes. A recent prospective cohort study sought to examine whether long-term adherence to a plant-based diet reduced the risk of recurrence and mortality in women diagnosed with breast cancer with a median follow-up of 9.5 years.^12^ In this study, a plant-based diet was associated with reduced all-cause and non–breast cancer mortality (HR = 0.83, 95% CI, 0.71-0.98) but no effect was seen in breast cancer mortality. A meta-analysis of randomized controlled trials and cohort studies found that adherence to a low-fat, high-quality, prudent diet was associated with reduced risk of non–breast cancer-related deaths in breast cancer survivors and that adherence to a Western diet before and after diagnosis increased risk of all-cause mortality.^13^ Similarly, the Women’s Health Initiative revealed that adherence to a lower-quality diet after breast cancer diagnosis, as assessed through the Healthy Eating Index score, was associated with an increased risk of death from breast cancer (HR = 1.66, 95% CI, 1.09-2.52).^14^ The Nurses’ Health Study assessed the associations of postdiagnosis fruit and vegetable consumption in breast cancer patients (stage I-III) and found that women in the highest quintile of fruit and vegetable consumption had significantly lower all-cause mortality relative to women in the lowest quintile (HR = 0.82, 95% CI, 0.71-0.94), but no association with breast cancer–specific mortality was detected.^15^

Numerous retrospective studies explore the relationship between adiposity or waist circumference on breast cancer mortality. The Cancer Prevention Study-II Nutrition Cohort examined patients living with breast cancer with local or regional breast cancer and found that pre- and postdiagnosis BMI was associated with a higher risk of breast cancer-specific mortality (HR = 1.27, 95% CI, 1.14-1.41) in women aged ≥65 years independent of comorbidities, although this association did not hold for women aged <65 years.^16^ A more recent systematic review of prospective studies including 519 544 patients living with breast cancer examined the associations of general and central adiposity (BMI and waist circumference, respectively) on all-cause mortality, breast cancer mortality, and cancer recurrence.^17^ BMI of ≥30 kg/m^2^ was associated with increased risk of all-cause mortality (relative risk (RR) = 1.21, 95% CI, 1.15-1.27), breast cancer-specific mortality (RR = 1.22, 95% CI, 1.13-1.32), breast cancer recurrence (RR = 1.19, 95% CI, 1.06-1.18), and distant recurrence (RR 1.19, 95% CI, 1.11-1.28), regardless of menopausal status. Central adiposity analyses were less striking due to large heterogeneity between studies. Waist circumference was associated with all-cause mortality, but no association was detected between central adiposity and breast cancer-specific mortality. A large weight gain defined as >10% from prior to or at the time of diagnosis to postdiagnosis was associated with increased all-cause mortality (RR = 1.28, 95% CI, 1.09-1.50), breast cancer-specific mortality (RR = 1.40, 95% CI, 1.08-1.80), and breast cancer recurrence (RR = 1.30, 95% CI, 1.10-1.54). In subtype analysis for BMI, interestingly, associations were weaker for patients with estrogen receptor-negative (ER−) disease and no association existed for patients with triple-negative tumor types.^17^ These findings may be attributed to the peripheral synthesis of estrogen in adipose tissue and its downstream effects on hormone-sensitive tumor progression.

On the basis of these studies, a low-fat, whole food, plant-forward diet with minimally processed food rich in vegetables, fruits, whole grains, legumes, nuts, and seeds is likely optimal for the breast cancer survivor. The World Cancer Research Fund/American Institute for Cancer Research (WCRF/AICR) has offered an expert report on diet, nutrition, and physical activity for patients living with breast cancer for preventing cancer and generally recommends a healthful diet targeted toward proper weight management (Table 2).^18^ Detailed dietary recommendations from the WCRF/AICR include the consumption of a diet rich in whole grains, vegetables, fruits, and beans, limitation of red and processed meats, limitation of sugar-sweetened beverages, and limitation of alcohol. The WCRF/AICR also recommends against the use of supplements for nutrition with an emphasis on obtaining nutritional needs from diet alone (Table 2). Socioeconomic, geographic, and educational factors contribute to dietary patterns in our diverse patient populations and must be considered by oncologists as they seek to counsel patients on healthful eating practices.

**Table 2.** The 2018 WCRF/AICR cancer prevention nutrition recommendations.

Recommendations	Details
Eat a diet rich in whole grains, vegetables, fruits, and beans (legumes and lentils)	Consume a diet that provides at least 30 g/day of fiber from food sources Include in more meals foods containing whole grains, nonstarchy vegetables, fruits, and pulses (legumes) such as beans and lentils Eat a diet high in all types of plant foods including at least 5 portions or servings (at least 400 g or 15 oz in total) of a variety of nonstarchy vegetables and fruits every day If starchy roots and tubers are staple foods, eat nonstarchy vegetables (legumes) regularly if possible
Limit consumption of processed foods high in fat, starches, or sugars	This includes limiting the consumption of fast foods, many prepared dishes, snacks, bakery foods and desserts; and candy
Limit consumption of red meat and avoid processed meat if possible	Limit consumption of red meat to no more than about 3 portions per week. One portion is approximately 4-6 oz cooked weight of red meat Consume very little, if any, processed meat
Limit consumption of sugar-sweetened drinks	Drink mostly water and unsweetened drinks Do not consume sugar-sweetened drinks
Limit alcohol consumption	For cancer prevention, it is best not to drink alcohol at all
Do not use supplements for cancer prevention	High-dose dietary supplements are not recommended for cancer prevention Aim to meet nutritional needs through diet alone

## Social Connections

The benefits of a social support system on human health, including mortality, quality of life, and symptom burden, have been characterized in numerous studies.^19^ A challenge to investigating causal relationships between social structure and disease process lies in defining social support, as numerous measurable psychosocial variables play into defining an individual’s social support system. Broadly, social support can be defined as structural or quantitative support (ie, size of a social network, presence/absence of a confidant) and functional or qualitative support (ie, perceived positive support, strength of ties). Both quantity and quality of social support are thought to contribute to quality of life and disease outcomes.

With regard to cancer, social connections have long been suggested to have direct and indirect effects on emotional adjustment to a cancer diagnosis and overall patient well-being during the cancer journey.^20-23^ Importantly, a body of work supports a correlative role for social integration and support in improved breast cancer prognosis and prevention of recurrence.^24-28^ In a prospective study, women with invasive breast cancer who experience prediagnosis social isolation have a 66% increase in risk of all-cause mortality (HR = 1.66; 95% CI, 1.04-2.65) and a 2-fold increase in breast cancer mortality compared with women who are socially integrated (HR = 2.14; 95% CI, 1.11-4.12).^29^ Results from this trial were adjusted for numerous covariates including breast cancer stage and parity. Specifically, Kroenke et al found that a lack of close relatives, friends, or living children was each associated with increased breast cancer mortality.^29^ In a systematic review examining cancer progression and indices of social support, breast cancer emerged as the only malignancy with sufficient evidence to demonstrate a positive relationship between social capital and cancer outcome. The meta-analyses revealed that structural social support defined as the total number of people in a patient’s personal social sphere, organizational involvement, frequency of social contact, and availability of a confidant were associated with significantly improved disease-free survival defined as calculated time to recurrence or death, whichever came first.^23^ Nausheen et al showed that a low perception of emotional or functional support was associated with a higher risk of all-cause mortality in women with stage II-IV breast cancers.^23,30^

Dr. Vivek Murthy, the Surgeon General of the United States, is actively bringing light to what is being termed a “loneliness epidemic” with significant public health implications for cancer. Specifically, any degree of loneliness has been found to be associated with a higher risk of mortality in patients living with cancer.^31^ Given the positive relationship between breast cancer survival and robust social support, it behooves survivorship programs to seek opportunities to assess loneliness, and foster social connections during and after cancer treatment, particularly for vulnerable and isolated patients lacking family or partnerships.

## Sleep

Epidemiological studies have demonstrated the close relationship between sleep quality and overall health outcomes in the general population. In the prediagnosis setting, chronic circadian rhythm dysregulation has been shown to increase the risk of breast cancer,^32,33^ supporting a role for sleep in the prevention of breast cancer. In addition to incidence, prognosis may also be affected by sleep. The Women’s Health Initiative found that, among breast cancer survivors, short sleep of ≤5 hours per night prior to diagnosis was associated with reduced breast cancer survival relative to women who slept 7-8 hours per night.^34^ Up to 70% of patients living with breast cancer and survivors report sleep disturbances including difficulty falling asleep, staying asleep, and overall compromised sleep time,^35^ likely related to exacerbation of menopausal symptoms after chemotherapy or endocrine therapy, anxiety of cancer recurrence, pain, and depression.

Only a handful of studies have investigated whether sleep duration can affect breast cancer recurrence after diagnosis. In the multicenter Women’s Healthy Eating and Living Study, Marinac et al asked whether self-reported baseline sleep duration or changes in sleep patterns were associated with breast cancer prognosis among survivors of early-stage breast cancer.^36^ In women who reported sleeping 7-8 hours per night at baseline, sleeping ≥9 hours/night was associated with a striking 48% increased risk of breast cancer recurrence (HR = 1.48; 95% CI, 1.01-2.13) and a 52% greater risk of breast cancer-specific mortality (HR = 1.52; 95% CI, 1.09-2.13), and a 43% increase in all-cause mortality (HR = 1.43; 95% CI, 1.07-1.92). These findings only held true when sleep of ≥9 hours per night was reported as an increase from baseline. Interestingly, the effects of sleep duration change on breast cancer prognosis were independent of changes in lifestyle factors, physical and mental health, and co-morbidity status.

Given the high prevalence of insomnia in patients living with cancer, a recent comprehensive systematic review and meta-analysis was conducted to evaluate the efficacy of pharmacological and nonpharmacological sleep interventions for adult patients living with cancer and survivors.^37^ Interestingly, cognitive behavioral therapy for insomnia (CBT-I) was found to be the most effective intervention identified and should be considered a first-line treatment for insomnia in patients with breast cancer.^37^

Taken together, a well-regulated circadian cycle and approximately 7-8 hours of quality sleep appear to reduce the risk of breast cancer and improve prognosis in women before and after breast cancer diagnosis. Highly variable sleep (<5 hours or>9 hours) may increase the risk of recurrence and be associated with higher mortality.

## Stress management

Psychological stress has been directly linked to increased cardiovascular, immune, psychiatric, and metabolic disease through the modulation of neuroendocrine and immune pathways.^38^ Epidemiological and preclinical studies suggest that chronic uncontrolled stress, through similar pathways, may play a role in cancer initiation, progression, and metastasis, and could negatively affect therapy response by weakening the immune system.^39^ Patients living with breast cancer and survivors are often subject to the stress of difficult treatments and fear of recurrence or progression. Low socioeconomic support can exacerbate this stress and the body’s subsequent response. Stress management interventions have consistently been shown to reduce serum cortisol and inflammatory cytokine production, increase relaxation, lower cancer-specific anxiety, and generalized anxiety in patients with breast cancer.^40,41^

The efficacy of stress reduction interventions in the prevention of breast cancer recurrence is controversial, as data are limited and conflicting. Two randomized controlled trials have demonstrated a significant reduction in breast cancer recurrence in patients receiving psychological intervention, particularly after surgical resection of breast cancer. Anderson et al randomized patients with breast cancer into either a treatment arm with small group psychologist-led interventions or a control arm where patients were only assessed and followed for 11 years. Patients in the intervention arm were found to have a reduced risk of breast cancer recurrence and death compared with patients in the control arm.^42^ Psychological intervention also reduced the risk of all-cause mortality. In a separate study, postoperative breast cancer patients between stages 0 and IIIb were randomized to a 10-week group-based cognitive behavioral stress management therapy arm or to a control arm where patients attended a 1-day psychoeducational seminar.^43^ Patients were followed up for 8-15 years and relative to the control group, patients who underwent cognitive behavioral stress management had a reduction in all-cause mortality. When restricted to women with invasive disease, the cognitive behavioral stress management therapy arm had significantly reduced breast cancer mortality and overall increased disease-free interval.^43^ Further studies are needed to elucidate a clear survival benefit for psychological interventions in breast cancer.

What is undisputed, however, is the benefit of psychological care and stress reduction in reducing symptoms of anxiety and depression, increasing self-efficacy, improving adherence to therapy, reducing fatigue, and improving social function among patients with breast cancer.^44^ On the basis of the prevalence of psychological stress in patients living with cancer, the ASCO recognizes psychological care as a facet of cancer care by offering practice guidelines, recommending that all patients with cancer are screened for symptoms of depression and anxiety during the trajectory of care.^45^ Moreover, cancer centers are increasingly implementing group interventions, built-in peer support, and referring patients for psychiatric treatment as the effect of psychological stress on survivorship quality of life is recognized.

## Avoidance of risky substances

Landmark studies in the 1980s published in the *New England Journal of Medicine* found an association between moderate alcohol intake (3-9 drinks per week) and increased risk of breast cancer.^46,47^ As breast cancer survivorship continues to improve, a focus on the study of modifiable risk factors in the survivor population has grown. The Life After Cancer Epidemiology (LACE) study followed alcohol consumption in 1897 patients with early-stage breast cancer after diagnosis with an average follow-up period of 7.4 years.^48^ The study demonstrated that consumption of ≥6 g of alcohol per day (equivalent to as few as 3-4 alcoholic drinks per week) relative to women who did not consume alcohol was associated with increased risk of breast cancer recurrence (HR = 1.35, 95% CI, 1.00-1.83), and death due to breast cancer (HR = 1.51, 95% CI, 1.00-2.29). Notably, subgroup analyses revealed that the effect was greater in postmenopausal and overweight or obese women. Interestingly, alcohol intake was not associated with all-cause mortality or non–breast cancer-related death, suggesting a direct effect on breast cancer pathophysiology.

Similar to alcohol, active and passive smoking of tobacco has been linked to increased risk for breast cancer in retrospective case–control studies and prospective cohort studies.^49^ In the survivorship setting, data from the After Breast Cancer Pooling Project were pooled and authors sought to determine whether former smokers had increased risk of recurrence or mortality.^50^ Relative to never-smokers, former smokers with less than 20-pack-year history of exposure had no increased risk on any outcome examined. However, former smokers with 20 to 34.5 pack-year history had a 22% increased risk of breast cancer recurrence (HR = 1.22, 95% CI, 1.01-1.48) and a 26% increase in all-cause mortality (HR = 1.26, 95% CI, 1.07-1.48). Effects appeared dose dependent as former smokers with ≥35 pack-year history had a 37% increased risk of recurrence (HR = 1.37, 95% CI, 1.13-1.66), a 54% increased risk of breast cancer mortality (HR = 1.54, 95% CI, 1.24-1.91), and a 68% increase in all-cause mortality (HR = 1.68, 95% CI, 1.44-1.96). Current smoking increased the probability of breast cancer recurrence by 41% (HR = 1.41; 95% CI, = 1.16-1.71), increased breast cancer mortality by 60% (HR = 1.61, 95% CI, 1.28-2.03), and doubled the risk of all-cause mortality (HR = 2.17, 95% CI, 1.85-2.54).

These data are striking and point toward a need for patient education during clinic visits about the harm of alcohol and tobacco, and perhaps importantly, formal programming for breast cancer survivors struggling to quit substances that are causing them harm.

## Discussion

Oncologists are regularly managing issues of survivorship during prolonged periods of active surveillance. The number of breast cancer survivors continues to increase, thus the topic of prevention of breast cancer recurrence is a central theme in many outpatient oncology visits. Patients often seek counsel on tangible ways to reduce their risk of breast cancer recurrence and are met with generic suggestions to live healthfully. Lifestyle medicine approaches have been a central theme in the prevention of breast cancer^51^; however, until now no such literature review has outlined the known benefits of each of each facet of lifestyle medicine in the prevention of breast cancer recurrence. Overwhelmingly, the clinical trials presented in here point toward the substantial benefit of adopting the 6 pillars of lifestyle medicine in a comprehensive cancer survivorship program to improve disease-free survival and all-cause mortality in patients living with breast cancer. Socially integrated patients and patients who undergo cognitive behavioral therapy for the management of stress tend to live longer and have a better-perceived quality of life than patients who are isolated or experiencing heightened anxiety. Collectively, the literature is clear that ongoing smoking and consumption of >6 g of alcohol per day as a breast cancer survivor significantly increases the risk of death from breast cancer. Patients struggling with addiction are owed a poignant discussion and opportunity to enroll in treatment. Finally, prospective and retrospective studies have shown that the maintenance of a healthy body weight and adequate nutrition through dietary interventions, aerobic and resistance exercise, and balanced circadian sleep, particularly in postmenopausal women, improve breast cancer survival outcomes. In summary, evidence exists for the benefit of all 6 of the pillars of lifestyle medicine in breast cancer survival.

Comprehensive cancer care should include optimizing a patient for survivorship by bolstering social connectivity, enrollment in diet, exercise, and stress management programming, sleep training, and addiction treatment. We recognize that an oncologist cannot be a psychologist, personal trainer, dietician, or a member of a patient’s inner circle of support. Therefore, it behooves cancer centers to consider the development of organized survivorship programs aimed at giving patients the best possible chance of preventing cancer recurrence and promoting quality of life. On the basis of a growing body of literature, the enrollment of breast cancer survivors in formal lifestyle medicine programs with coaching, education, and lifestyle interventions would improve and save lives.

## Data Availability

No new data were generated or analyzed in this manuscript.
